# Automatic assessment of laparoscopic surgical skill competence based on motion metrics

**DOI:** 10.1371/journal.pone.0277105

**Published:** 2022-11-02

**Authors:** Koki Ebina, Takashige Abe, Kiyohiko Hotta, Madoka Higuchi, Jun Furumido, Naoya Iwahara, Masafumi Kon, Kou Miyaji, Sayaka Shibuya, Yan Lingbo, Shunsuke Komizunai, Yo Kurashima, Hiroshi Kikuchi, Ryuji Matsumoto, Takahiro Osawa, Sachiyo Murai, Teppei Tsujita, Kazuya Sase, Xiaoshuai Chen, Atsushi Konno, Nobuo Shinohara

**Affiliations:** 1 Graduate School of Information Science and Technology, Hokkaido University, Sapporo, Japan; 2 Department of Urology, Hokkaido University Graduate School of Medicine, Sapporo, Japan; 3 Hokkaido University Clinical Simulation Center, Hokkaido University Graduate School of Medicine, Sapporo, Japan; 4 Department of Mechanical Engineering, National Defense Academy of Japan, Yokosuka, Japan; 5 Department of Mechanical Engineering and Intelligent Systems, Tohoku Gakuin University, Tagajo, Japan; 6 Graduate School of Science and Technology, Hirosaki University, Hirosaki, Japan; Vels Institute of Science, Technology and Advanced Studies (VISTAS), MALAYSIA

## Abstract

The purpose of this study was to characterize the motion features of surgical devices associated with laparoscopic surgical competency and build an automatic skill-credential system in porcine cadaver organ simulation training. Participants performed tissue dissection around the aorta, dividing vascular pedicles after applying Hem-o-lok (tissue dissection task) and parenchymal closure of the kidney (suturing task). Movements of surgical devices were tracked by a motion capture (Mocap) system, and Mocap-metrics were compared according to the level of surgical experience (experts: ≥50 laparoscopic surgeries, intermediates: 10–49, novices: 0–9), using the Kruskal-Wallis test and principal component analysis (PCA). Three machine-learning algorithms: support vector machine (SVM), PCA-SVM, and gradient boosting decision tree (GBDT), were utilized for discrimination of the surgical experience level. The accuracy of each model was evaluated by nested and repeated k-fold cross-validation. A total of 32 experts, 18 intermediates, and 20 novices participated in the present study. PCA revealed that efficiency-related metrics (e.g., path length) significantly contributed to PC 1 in both tasks. Regarding PC 2, speed-related metrics (e.g., velocity, acceleration, jerk) of right-hand devices largely contributed to the tissue dissection task, while those of left-hand devices did in the suturing task. Regarding the three-group discrimination, in the tissue dissection task, the GBDT method was superior to the other methods (median accuracy: 68.6%). In the suturing task, SVM and PCA-SVM methods were superior to the GBDT method (57.4 and 58.4%, respectively). Regarding the two-group discrimination (experts vs. intermediates/novices), the GBDT method resulted in a median accuracy of 72.9% in the tissue dissection task, and, in the suturing task, the PCA-SVM method resulted in a median accuracy of 69.2%. Overall, the mocap-based credential system using machine-learning classifiers provides a correct judgment rate of around 70% (two-group discrimination). Together with motion analysis and wet-lab training, simulation training could be a practical method for objectively assessing the surgical competence of trainees.

## Introduction

The traditional apprenticeship model of surgical education: “see one, do one, teach one”, has now become less acceptable. Along with 1: the widespread dissemination of laparoscopic and robotic surgeries that necessitate specific surgical skills, 2: regulation of working hours, and 3: social demand for safer surgery, laboratory-based skill training has been utilized in a wide range of surgical disciplines. In the authors’ previous study, a low-cost wet-lab model using cadaveric swine organs, including tissue dissection around the aorta and renal parenchymal closure, was developed, and training drills showed good construct validity [1]. Furthermore, a novel motion capture (Mocap) based measurement system that consists of 6 infrared cameras was developed. This system simultaneously tracked the movements of multiple surgical instruments, and identified the motion characteristics according to the level of laparoscopic surgical experiences in wet-lab training [2]. For example, in a tissue dissection task, a shorter path length and faster velocity/acceleration/jerk were observed for scissors and a Hem-o-lok applier in experts (≥50 laparoscopic surgeries), and in experts with ≥100 cases, scissors moved more frequently in the close zone (0≤ to <2 cm from aorta) than those with 50–99 cases [3].

To ensure that trainees are ready to perform surgery, skills assessments are becoming more important, and they are traditionally performed manually by observing training tasks on site or video footage according to global skill assessment tools, such as “Objective Structured Assessment of Technical Skills (OSTAS)” or “Global Operative Assessment of Laparoscopic Skills (GOALS)”, that usually markedly increase workloads of mentors [4,5]. Regarding automated assessment, several studies reported promising results. For example, Allen B et al. reported that in 30 participants (4 experts and 26 novices) performing the three drills of peg transfer, pass rope, and cap needle, instrument movements were captured by two electromagnetic sensors, and the support vector machines (SVMs) yielded >90% competency-prediction based on the motion metrics [6]. However, prior studies utilized very simple training drills such as “peg transfer”, “pattern cutting”, or “suturing” that involved artificial materials. Ideally, more complex drills should be included in credential processes before trainees perform actual surgery.

In the present study, in order to gain further insight into movement features of experts, data collection was expanded to include laparoscopic surgeons other than urologic surgeons. Using motion metrics of surgical instruments and several machine-learning techniques, we aimed to automatically assess surgical competence in porcine cadaver organ simulation training.

## Materials and methods

This study was approved by the Ethical Review Board for Life Science and Medical Research, Hokkaido University Hospital (No. 018–0257). The initial results based on the current Mocap based measurement system among urologic surgeons, a junior trainee, and medical students (1^st^ data collection: n = 45, between December 2018 and February 2019) have been published [3]. In order to gain further insights into Mocap characteristics of experts and develop an efficient training model, the measurement experiments including general and gynecologic surgeons (2^nd^ data collection: between the end of May 2019 and September 2019) were extended. In the 2^nd^ data collection, participants performed tissue dissection around a porcine aorta (Task 1) and renal parenchymal closure (Task 3), while needle driving in renal parenchyma (Task 2) was not performed because of the similar outcomes of Mocap metrics between Tasks 2 and 3 divided by the level of surgical experiences in the authors’ previous study [3]. Overall, a total of 70 participants voluntarily took part in 89 training sessions of Tasks 1 and 3 during the total study period (19 participants overlapped between the 1st and 2nd data collections). Written informed consent was obtained regarding the use of their data for research.

The details of the present training tasks were previously reported [3]. In brief, porcine cadaveric organs were placed in a training box (Endowork ProⅡ®, Kyoto Kagaku, Japan). During the training, one of the 4 authors (TA, MH, JF, and NI) assumed the role of a scopist, using an endoscopic camera system (VISERA Pro Video System Center OTV-S7Pro, Olympus, Japan). In Task 1, participants were asked to complete tissue dissection around the aorta, dividing encountered mesenteric vessels after applying Hem-o-lok. In Task 3, using a 15-cm 2–0 CT-1 VICRYL® thread, participants were asked to make three square single-throw knots at 2 different sites on a kidney. All training was video-recorded for later analyses. Demographic data and prior experience of laparoscopic surgeries were also collected after the training.

### Motion capture analysis

**Fig 1:**
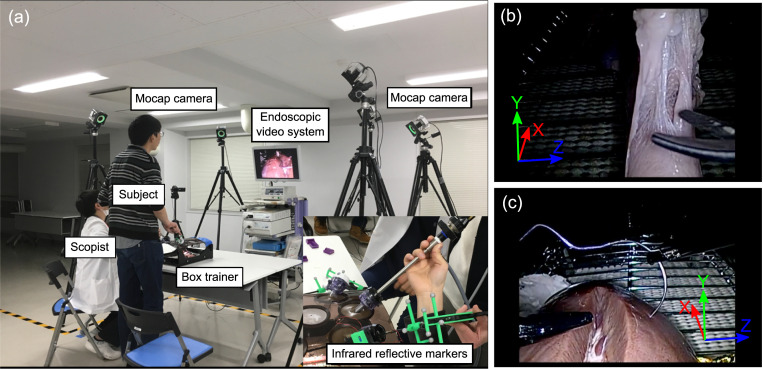
Photographs of the simulation training. (a): The Mocap-based measurement system, which consisted of 6 infrared cameras (OptiTrack Prime 41, NaturalPoint Inc., USA), simultaneously tracked infrared reflective markers attached to multiple surgical instruments during each training task. (b): Task 1, a view of tissue dissection. (c): Task 3, a view of needle driving.

The details of the present Mocap based measurement system were previously published [2]. In brief, the measurement system simultaneously tracked multiple surgical instruments by 6 infrared cameras (OptiTrack Prime 41, NaturalPoint Inc., USA). Infrared reflective marker sets with a different pattern were connected to handles of each instrument so that they could be traced individually regardless of exchanges of instruments. The tip trajectory was calculated based on the position of the tip and handle. In order to reduce the noise, the track of the tip of instruments (*x*_*i*_, *y*_*i*_, and *z*_*i*_) was smoothed with a Savitzky-Golay filter [7], and its derivatives ($\frac{d^{j}x_{i}}{dt^{j}},\frac{d^{j}y_{i}}{dt^{j}},\;and\;\frac{d^{j}z_{i}}{dt^{j}}\;(j=1\;to\;3)$) were also calculated by the filter. In the 2^nd^ data collection, in order to measure the grasping force and position of grasping forceps, grasping forceps with strain gauges were utilized in Task 1, although it was not a focus of the current study. Fig 1 shows the present Mocap-based measurement system, and endoscopic views of training tasks.

### Analysis and statistics

In order to characterize the motion features of surgical devices associated with laparoscopic surgical competency, motion metrics that represent kinematic features of the surgical instrument were calculated. S1 Table summarizes the definition of Mocap metrics. In addition to the metrics already reported in the authors’ previous study [3], 10 metrics were newly calculated, based on hypotheses generated during the authors’ video review process and previous papers: bimanual dexterity (BD), ratio of frequency of opening/closing both forceps (ROB), ratio of path length for both hands (RPLB), average distance between both forceps when opening/closing (ADBO), average distance between both forceps (ADB), depth path length (DPL), depth velocity (DV), average gripper rotation angle (AGRA), average attitude angle (Roll, Pitch, Yaw), angular length (AL, Roll or Pitch/Yaw), and working area (WA). For example, regarding ADB, the authors hypothesized that it would become closer in experts in the suturing/knotting task because of their efficient movements. Because of good depth perception, it was hypothesized that DPL would be shorter, and DV would become faster in experts was built in both tasks. Regarding WA, it was also hypothesized that WA of experts would become smaller than that of novices because experts manipulate surgical devices in the area close to the objectives in both tasks. Regarding AL-Roll, the hypothesis was that the sum of changes in the attitude angle of an instrument around the sheath axis would become smaller in experts due to better visual spatial ability.

The following is an outline of the present analyses:

1. Mocap-metrics were compared according to previous laparoscopic surgical cases (experts: ≥50 surgeries, intermediates: 10–49, novices: 0–9). The Kruskal-Wallis test was utilized to evaluate differences among the three groups. The Mann-Whitney U test was also utilized for paired comparison, if differences among the three groups were significant.
2. Using the Mocap-metrics with significant differences among the 3 abovementioned groups, principal component analysis (PCA) was performed, a data reduction technique, in order to identify the motion characteristics associated with surgical competency intuitively.
3. Finally, three machine-learning algorithms: Support Vector Machine (SVM), Principal component analysis-SVM (PCA-SVM), and Gradient Boosting Decision Tree (GBDT), were utilized for discrimination of the surgical experience level based on Mocap-metrics. The details of these algorithms are described in S2 Table.

Before inputting all Mocap indices to these algorithms, robust Z-score normalization was conducted for scaling the data while reducing the effects of outliers. The robust Z score, *z*_*i*_, for data, *x*_*i*_, can be calculated as follows:

$$z_{i}=\frac{x_{i}-x_{m}}{NIQR}.$$ (1)

Here, *x*_m_ is the median for data ***x***, and *NIQR* is the normalized interquartile range, calculated as *NIQR* = 0.7414⋅*IQR* (*IQR* = Interquartile range).

The model was validated using nested and repeated k-fold cross-validation, which is a combined method of nested k-fold cross-validation and repeated cross-validation. This method enables robust verification that is not affected by randomness. S1 Fig shows that the data flow of the validation process. All procedures related to machine learning were done using Scikit-learn, a machine-learning library for python [8]. The machine-learning library “LightGBM” was also used to build a model of GBDT [9]. The accuracies of machine-learning models were compared by Friedman’s test. The Wilcoxon signed rank sum test was also utilized to assess the differences in paired comparison. Friedman’s test and the Wilcoxon signed rank sum test were performed using JMP 14 (SAS, Japan), and PCA was performed using R (Ver. 3.6.0).

## Results

Table 1 shows a summary of participants’ backgrounds. Urologic surgeons were dominant (n = 45), followed by gastroenterological surgeons (n = 9), medical students (n = 9), junior residents (n = 4), and gynecologic surgeons (n = 3). The experiences of laparoscopic surgery were: 0–9: n = 20, 10–49: n = 18, 50–99: n = 7, 100–499: n = 18, and ≥500: n = 7. As described above, 19 joined the training multiple times, which resulted in a total of 89 training sessions.

**Table 1:** Participants’ backgrounds.

	n = 70
**Background**	Urologic surgeon, n = 45
	Gastroenterological surgeon, n = 9
	Gynecologic surgeon, n = 3
	Junior resident, n = 4
	Medical student, n = 9
**Age, years**	Median 35 (range, 23–57)
**Sex**	Male/Female = 61/9
**Experience of laparoscopic surgery**	0–9, n = 20
	10–49, n = 18
	50–99, n = 7
	100–499, n = 18
	≥500, n = 7
**Dominant hand**	Right/left = 67/3

S3 Table summarizes Mocap metrics according to previous surgical experiences. Overall, there were significant differences in speed-related metrics including velocity, acceleration, and jerk in scissors, Hem-o-lok clip applier, and bilateral needle holders, and significant differences in efficiency-related metrics including the operative time and path length in all devices among the three groups. These observations were in line with our previous study. Regarding the new metrics, for example, BD (in both tasks), DPL in grasping forceps, scissors, Hem-o-lok, and right/left needle holders, and AL-Pitch/Yaw in grasping forceps, scissors, clip applier, and right/left needle holders showed significant differences among the 3 groups.

**Fig 2:**
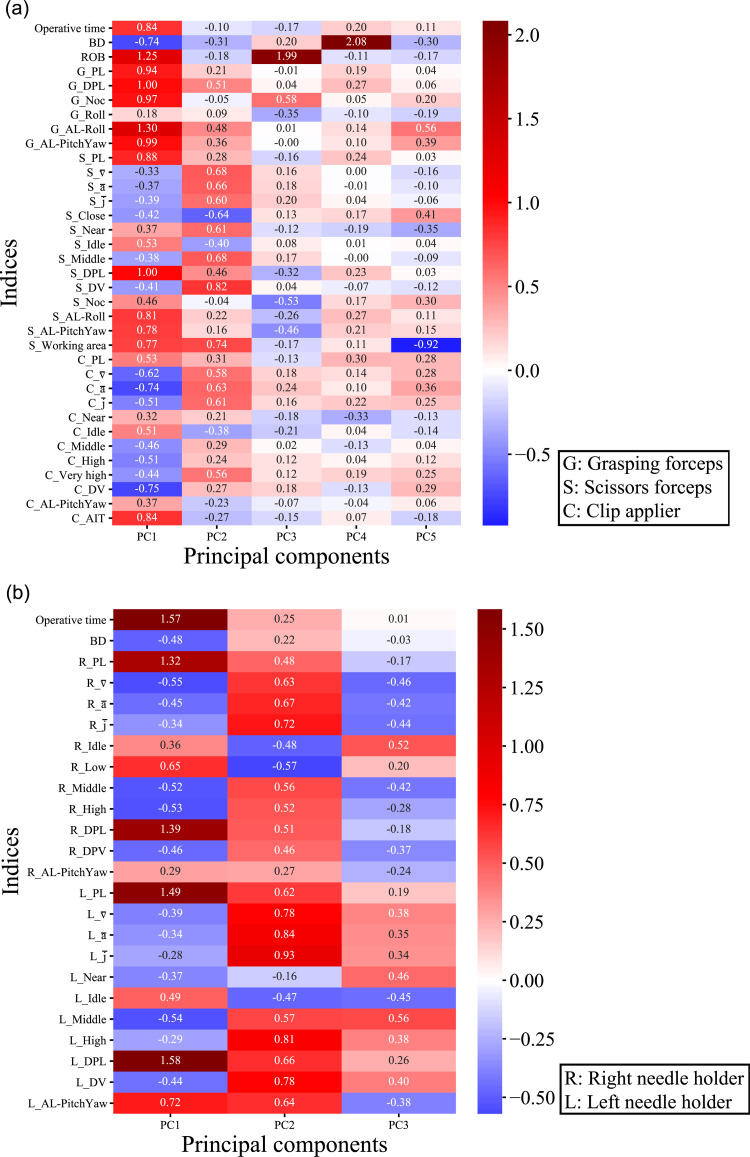
PCA loading plot. (a): In task 1, efficiency-related metrics (e.g., ROB, G_DPL, G_AL-Roll, and S_DPL) significantly contributed to PC1, and speed-related parameters in surgical devices manipulated by the right hand (e.g., $S\_\bar{v}$, $C\_\bar{v}$, $S\_\bar{a}$, $C\_\bar{a}$, $S\_\bar{j}$, $C\_\bar{j}$, and S_DV) significantly contributed to PC2. Regarding PC3, PC4, and PC5, ROB, BD, and S_Working area largely contributed to each PC, respectively. (b): In task 3, efficiency-related parameters in both needle holders (e.g., operative time, R_PL, L_PL, R_DPL, and L_DPL) largely contributed to PC1, and speed-related parameters, especially of a left needle holder (e.g., $L\_\bar{j}$, $L\_\bar{a}$, L_High, $L\_\bar{v}$, and $R\_\bar{j}$), significantly contributed to PC2.

Fig 2 shows a PCA loading plot of both tasks (a: Task 1, b: Task 3). In Task 1 (Fig 2A), for example, ROB (loading coefficient = 1.25), G_DPL (loading coefficient = 1.00), G_AL-Roll (loading coefficient = 1.3), and S_DPL (loading coefficient = 1.0) largely contributed to PC1. In other words, efficiency-related metrics significantly contributed to PC1. Regarding PC2, $S\_\bar{v}$ (loading coefficient = 0.68), $C\_\bar{v}$ (loading coefficient = 0.58), $S\_\bar{a}$ (loading coefficient = 0.66), $C\_\bar{a}$ (loading coefficient = 0.63), $S\_\bar{j}$ (loading coefficient = 0.60), $C\_\bar{j}$ (loading coefficient = 0.61), and S_DV (loading coefficient = 0.82) largely contributed, which showed the significant contribution of speed-related parameters in surgical devices manipulated by the right hand. In PC3, PC4, and PC5, ROB (loading coefficient = 1.99), BD (loading coefficient = 2.08), and S_Working area (loading coefficient = -0.92) largely contributed to each PC, respectively. In Task 3 (Fig 2B), the operative time (loading value = 1.57), R_PL (loading coefficient = 1.32), L_PL (loading coefficient = 1.49), R_DPL (loading coefficient = 1.39), and L_DPL (loading coefficient = 1.58) largely contributed to PC1, which showed a significant contribution of efficiency-related parameters in both needle holders. Regarding PC2, $L\_\bar{j}$ (loading coefficient = 0.93), $L\_\bar{a}$ (loading coefficient = 0.84), L_High (loading coefficient = 0.81), $L\_\bar{v}$ (loading coefficient = 0.78), and $R\_\bar{j}$ (loading coefficient = 0.72) largely contributed, which showed the significant contribution of speed-related parameters, especially of a left needle holder.

**Fig 3:**
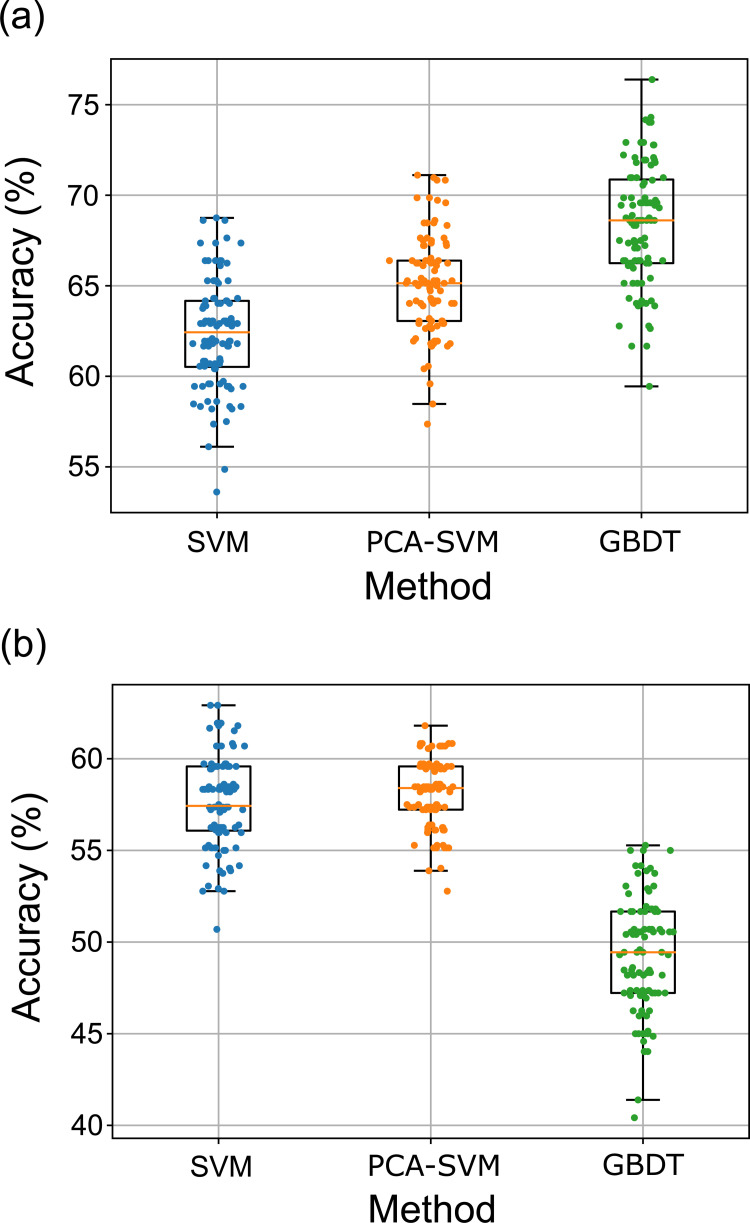
Accuracy of each classifier (3 grouping). **(**a): The GBDT method was superior to the other methods (median accuracy of 68.6%). (b): SVM and PCA-SVM methods were superior to the GBDT method (median accuracy of 57.4 and 58.4%, respectively). There was no significant difference between SVM and PCA-SVM.

**Fig 4:**
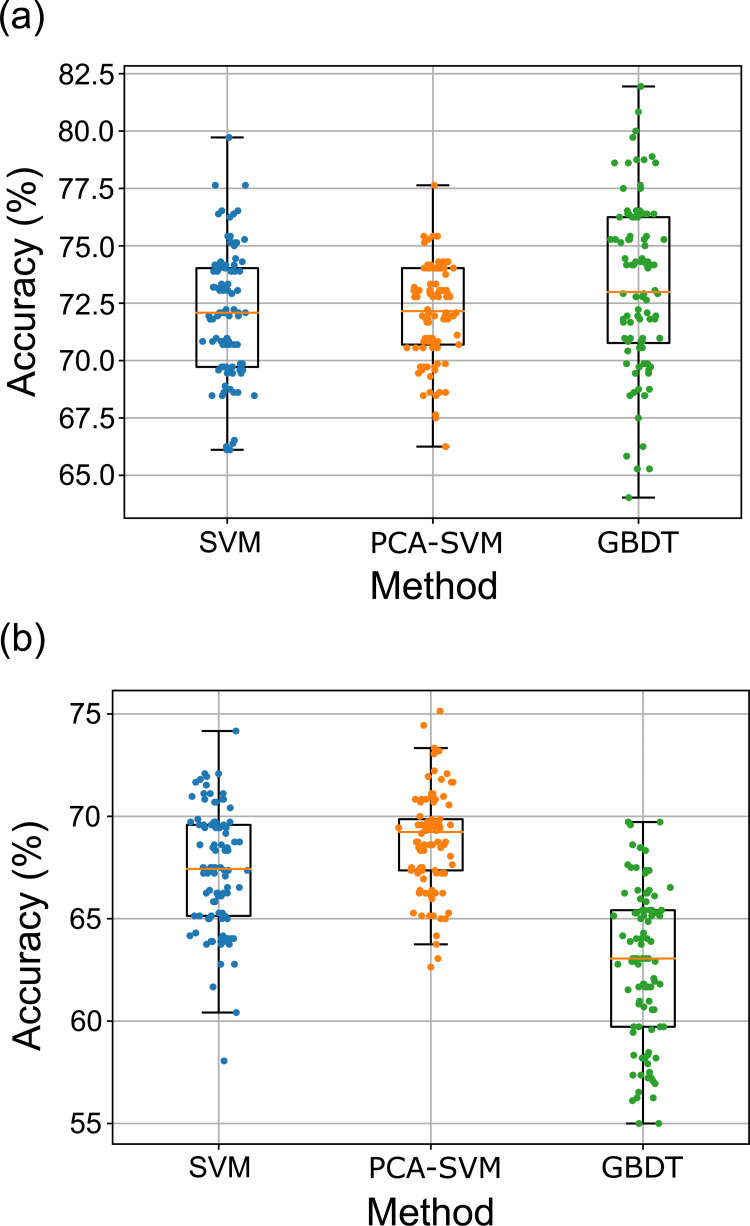
Accuracy of each classifier (2 grouping). (a): The GBDT method was superior to the other methods (median accuracy of 72.9%). (b): The PCA-SVM method was superior to the other methods (median accuracy of 69.2%).

Figs 3 and 4 and Table 2 show the performance results of each classifier under repeated and nested cross-validation, and comparative results for the three methods. Regarding the three-group discrimination (experts vs. intermediates vs. novices), in Task 1, the GBDT method was superior to the other methods. GBDT methods resulted in a median accuracy of 68.6% (Fig 3A and Table 2A). In Task 3, SVM and PCA-SVM methods were superior to the GBDT method (median accuracy of 57.4 and 58.4%, respectively, Fig 3B and Table 2A). There was no significant difference between SVM and PCA-SVM. Regarding the two-group discrimination (experts vs. intermediates/novices), GBDT methods resulted in a median accuracy of 72.9% in Task 1 (Fig 4A and Table 2B), and the PCA-SVM method resulted in a median accuracy of 69.2% in Task 3 (Fig 4B and Table 2B).

**Table 2:** Summary of comparative results of the three machine-learning models.

(a) Three-group discrimination (experts vs. intermediates vs. novices)							
	Median of accuracy (Interquartile range)			p-Value			
	PCA-SVM	SVM	GBDT	Friedman’s test	PCA-SVM vs. SVM	PCASVM vs. GBDT	SVM vs. GBDT
Task 1	0.6514 (0.6306–0.6639)	0.6243 (0.6052–0.6417)	0.6861 (0.6625–0.7087)	<0.0001	<0.0001	<0.0001	<0.0001
Task 3	0.5840 (0.5722–0.5958)	0.5743 (0.5608–0.5958)	0.4944 (0.4722–0.5167)	<0.0001	0.0741	<0.0001	<0.0001
(b) Two-group discrimination (experts vs. intermediates/novices)							
	Median of accuracy (Interquartile range)			p-Value			
	PCA-SVM	SVM	GBDT	Friedman’s test	PCA-SVM vs. SVM	PCASVM vs. GBDT	SVM vs. GBDT
Task 1	0.7215 (0.7069–0.7403)	0.7208 (0.6972–0.7403)	0.7299 (0.7076–0.7625)	0.0066	0.7496	0.0162	0.0017
Task 3	0.6924 (0.6736–0.6986)	0.6743 (0.6514–0.6958)	0.6306 (0.5972–0.6542)	<0.0001	<0.0001	<0.0001	<0.0001

SVM: Support Vector Machine, PCA-SVM: Principal Component Analysis-SVM, GBDT: Gradient Boosting Decision Tree.

## Discussion

In order to gain further insight into movement features of expert surgeons and automated skill assessment, the data collection, including urologic, gastroenterological, and gynecological surgeons who regularly performed laparoscopic surgery, was continued. As previously reported, the strength of this Mocap-based measurement system compared with previous studies is that all surgical devices can be tracked because of the arrangement of infrared reflective markers, and, therefore, the proposed model can be utilized in complex training tasks that require a range of surgical skills. In the present study, additional motion metrics not included in the previous study were newly calculated. According to past reports [10,11], BD was newly calculated, and it showed significant differences among the three groups in either task. DPL (grasping forceps, scissors, right/left needle holders) and DV (scissors, Hem-o-lok, right/left needle drivers) also showed significant differences, being in line with the hypothesis that good depth perception of experts results in shorter DPL and faster DV. In terms of AL-Roll and Al-Pitch/Yaw, which means the sum of changes in the attitude angle represented as Euler angles of an instrument, AL-Roll showed significant differences in grasping forceps and scissors, and Al-Pitch/Yaw in grasping forceps, scissors, Hem-o-lok, and right/left needle holders. Because Al-Pitch/Yaw means the sum of the angle change in the vertical plane of surgical devices, it is considered that large AL-Pitch/Yaw would mean both frequent angle adjustments and inefficient movements by less-experienced surgeons. Regarding smaller Al-Roll (sum of angle change along surgical device axis) in experts, it is considered that experts have good spatial ability that results in fewer angle adjustments (rotating a surgical device itself), and/or they use their index fingers efficiently to rotate the shaft of a surgical device, which was not reflected in the outcome of Al-Roll. Because this measurement system records detailed location records (30 Hz) of multiple surgical devices simultaneously, it enables subsequent analyses based on “surgical expertise”. In terms of PCA analyses, in a more generalized cohort including laparoscopic surgeons (urology, general surgery, and gynecology) and medical students, it is noted that efficiency-related metrics (e.g., ROB, G_DPL, G_AL-Roll, and S_DPL in Task 1, and operative time, R_PL, L_PL, R_DPL, and L_DPL in Task 3) significantly contributed to PC1, and speed-related metrics (e.g., $S\_\bar{v}$, $C\_\bar{v},S\_\bar{a}$, $C\_\bar{a}$, $S\_\bar{j},$ $C\_\bar{j}$, S_DV in Task 1, and $L\_\bar{j}$, $L\_\bar{a}$, L_High, $L\_\bar{v}$, and $R\_\bar{j}$ in Task 3) to PC2, being in line with a previous study.

Surgical skill evaluation facilitates surgical training and credentialing of competent surgeons. In this study, for automatic skill assessment, three classification methods: SVM, PCA-SVM, and GBDT, were evaluated. SVM has been frequently used for computer-based discrimination of surgeons’ expertise [6,12,13]. Because of the many feature values utilized in the present SVM model, which might lead to a risk of overfitting to the original data, the PCA-SVM method was also utilized in this study. In this method, classification of SVM is conducted after reducing the data dimension by PCA, and it is expected to prevent overfitting. Regarding GBDT that uses an ensemble of decision trees for target label prediction, it has also been frequently utilized in machine-learning research [14–16]. As summarized in Table 2, GBDT showed the best accuracy in Task 1(3-group discrimination: median accuracy of 68.6%, 2-group discrimination: 72.99%), and PCA-SVM in Task 3 (3-group discrimination: 58.4%, 2-group discrimination: 69.2%). In addition to PCA-SVM, SVM also revealed the best accuracy in 3-group discrimination of Task 3, although PCA-SVM should be a suitable method because PCA-SVM revealed the best accuracy in both discriminations.

It is considered that Task 1 includes a range of required skills (tissue traction/dissection, Hem-o-lok use, and division of vascular pedicle) compared with Task 3 (suturing/knotting), which should have resulted in better discrimination results in Task 1. Regarding the outcomes of two-group discrimination (experts vs. intermediates/novices), accuracy of around 0.7 in both tasks was similar to that in the study by Oropesa et al., wherein 42 participants performed 3 box trainer tasks (peg grasping task, task that requires placing three elastic bands through their corresponding posts, and coordinated peg transfer task), kinematic data were captured by the TrEndo tracking system, and linear discriminant analysis (LDA), SVM, and an adaptive neuro-fuzzy inference system (AN-FIS) were utilized to classify 42 participants according to prior surgical experience (>10 laparoscopic surgeries performed vs. <10) by leave-one-out cross-validation. The mean accuracy observed was 71% with LDA, 78.2% with SVM, and 71.7% with AN-FIS. Regarding the misclassification in the current study, it may reflect the limited correlation between the previous caseload and actual performance. For example, active medical students would perform dry box training of suturing/knotting regularly, which would result in better kinematic outcomes, especially in Task 3. As inherent limitations of each machine-learning algorithm, the configuration, training, and validation process might influence the misclassification. An external cohort is also necessary to validate this model, and larger training data and refinement of the algorithm is necessary in order to improve computer-based skill credentialing. Nevertheless, this study showed promising results in terms of automated skill credentialing based on kinematic tracking data of surgical devices in wet-lab training. As another direction, in order to provide more user-friendly feedback, we developed a machine-learning-based GOALS scoring system based on Mocap metrics, using the current dataset and recorded movies [17]. GOALS is an already validated and widely-used assessment tool for grading laparoscopic surgical skills, and consists of five items: depth perception, bimanual dexterity, efficiency, tissue handling, and autonomy [18]. It was reported that this system could evaluate surgeons’ skill with high accuracy (an error of approximately 1–2 points for a total score of 5–25 points). Taken together with the skill credential usage described in this paper, the authors believe that the motion data of instruments has promising value for surgical evaluation, which could provide valuable feedback to trainees, and mitigate the educators’ workload.

Limitations of this study include the small sample size, lack of an external validation cohort, and limited correlation between previous case volumes and surgical skills, as abovementioned. Furthermore, in order to assess the educational benefit of Mocap analyses and computer-based objective skill assessment in simulation training, developing a computer program for onsite feedback to trainees is needed.

## Conclusions

A Mocap-based credential system using machine-learning classifiers provides a correct judgment rate of around 70% (two-group discrimination). Together with motion analysis and wet-lab training, simulation training could be a practical method for objectively assessing the surgical competence of trainees. The next challenge is to give objective feedback based on mocap metrics to trainees immediately on-site, which could become an educational means together with mentors’ feedback.

## Supporting information

S1 Fig Overview of the dataflow in the model validation process of SVM/PCA-SVM/GBDT methods. NIQR = Normalized Interquartile Range, SVM = Support Vector Machine, PCA-SVM = Principal Component Analysis-SVM, GBDT = Gradient Boosting Decision Tree. Nested k-fold cross-validation consists of two validation processes: Outer Cross-validation (Outer CV) and Inner Cross-validation (Inner CV). Each cross-validation was conducted using 10-fold cross-validation. In each validation, the dataset were divided into 10 groups; 9 groups were used to train the model, and 1 group was for testing. The accuracy of the model was evaluated by repeating this process 10 times so that all groups were evaluated. Note that Inner CV was conducted using training data of Outer CV, although Outer CV was conducted using the entire dataset. The grid search for hyperparameter tuning was done in Inner CV. The best parameter that showed the highest accuracy among all candidate parameters was used to build the model for the outer CV. In this study, nested k-fold cross-validation was repeated 100 times with different dataset divisions.
(EPS)

S1 Table The definitions of measurement outcomes. (DOCX)

S2 Table Details of the 3 algorithms and candidate parameters for the grid search. (DOCX)

S3 Table Summary of statistical analysis of Mocap metrics. (DOCX)

## References

[1] Higuchi M, Abe T, Hotta K, Morita K, Miyata H, Furumido J, et al. Development and validation of a porcine organ model for training in essential laparoscopic surgical skills. Int J Urol. 2020; 27(10):929–938. doi: 10.1111/iju.14315 32743896 PMC7589398

[2] Ebina K, Komizunai S, Tsujita T, Sase K, Chen X, Higuchi M et al. Development and Validation of a Measurement System for Laparoscopic Surgical Procedures. SICE JCMSI. 2020; 13(4):191–200. 10.9746/jcmsi.13.191.

[3] Ebina K, Abe T, Higuchi M, Furumido J, Iwahara N, Kon M, et al. Motion analysis for better understanding of psychomotor skills in laparoscopy: objective assessment-based simulation training using animal organs. Surg Endosc. 2021; 35(8):4399–4416. doi: 10.1007/s00464-020-07940-7 32909201 PMC8263434

[4] Martin JA, Regehr G, Reznick R, MacRae H, Murnaghan J, Hutchison C, et al. Objective structured assessment of technical skill (OSATS) for surgical residents. Br J Surg. 1997; 84(2):273–278. doi: 10.1046/j.1365-2168.1997.02502.x 9052454

[5] Vassiliou MC, Feldman LS, Andrew CG, Bergman S, Leffondre K, Stanbridge D, et al. A global assessment tool for evaluation of intraoperative laparoscopic skills. Am J Surg. 2005; 190(1):107–113. doi: 10.1016/j.amjsurg.2005.04.004 15972181

[6] Allen B, Nistor V, Dutson E, Carman G, Lewis C, Faloutsos P. Support vector machines improve the accuracy of evaluation for the performance of laparoscopic training tasks. Surg Endosc. 2010; 24(1):170–178. doi: 10.1007/s00464-009-0556-6 19533237

[7] Savitzky A, Golay MJE. Smoothing and Differentiation of Data by Simplified Least Squares Procedures. Anal. Chem. 1964; 36(8):1627–1639. 10.1021/ac60214a047.

[8] Pedregosa F, Varoquaux G, Gramfort A, Michel V, Thirion B, Grisel O, el al. Scikit-learn: Machine Learning in Python. Journal of Machine Learning Research. 2011; 12:2825–2830.

[9] Ke G, Meng Q, Finley T, Wang T, Chen W, Ma W, et al. A highly efficient gradient boosting decision tree. 21 st Conference on Neural Information Processing Systems; 2017 Dec 4–9 Long Beach USA. Curran Associates, Inc. p. 3146–3154.

[10] Perez-Escamirosa F, Alarcon-Paredes A, Alonso-Silverio GA, Oropesa I, Camacho-Nieto O, Lorias-Espinoza D, et al. Objective classification of psychomotor laparoscopic skills of surgeons based on three different approaches. Int J Comput Assist Radiol Surg. 2020; 15 (1):27–40. doi: 10.1007/s11548-019-02073-2 31605351

[11] Hofstad EF, Vapenstad C, Bo LE, Lango T, Kuhry E, Marvik R. Psychomotor skills assessment by motion analysis in minimally invasive surgery on an animal organ. Minim Invasive Ther Allied Technol. 2017; 26(4):240–248. doi: 10.1080/13645706.2017.1284131 28635403

[12] Kowalewski KF, Garrow CR, Schmidt MW, Benner L, Muller-Stich BP, Nickel F. Sensor-based machine learning for workflow detection and as key to detect expert level in laparoscopic suturing and knot-tying, Surg Endosc. 2019; 33(11):3732–3740. doi: 10.1007/s00464-019-06667-4 30790048

[13] Oropesa I, Sanchez-Gonzaez P, Chmarra MK, Lamata P, Perez-Rodriguez R, Jansen FW, et al. Supervised classification of psychomotor competence in minimally invasive surgery based on instruments motion analysis. Surg Endosc. 2014; 28 (2):657–670. doi: 10.1007/s00464-013-3226-7 24122243

[14] Kawakami E, Tabata J, Yanaihara N, Ishikawa T, Koseki K, Iida Y, et al. Application of Artificial Intelligence for Preoperative Diagnostic and Prognostic Prediction in Epithelial Ovarian Cancer Based on Blood Biomarkers. Clin Cancer Res. 2019; 25(10):3006–3015. doi: 10.1158/1078-0432.CCR-18-3378 30979733

[15] Tahmassebi A, Wengert GJ, Helbich TH, Bago-Horvath Z, Alaei S, Bartsch R, et al. Impact of Machine Learning With Multiparametric Magnetic Resonance Imaging of the Breast for Early Prediction of Response to Neoadjuvant Chemotherapy and Survival Outcomes in Breast Cancer Patients. Invest Radiol. 2019; 54(2):110–117. doi: 10.1097/RLI.0000000000000518 30358693 PMC6310100

[16] Ricciardi C, Improta G, Amato F, Cesarelli G, Romano M. Classifying the type of delivery from cardiotocographic signals: A machine learning approach. Comput Methods Programs Biomed. 2020; 196:105712. doi: 10.1016/j.cmpb.2020.105712 32877811

[17] Ebina K, Abe T, Hotta K, Higuchi M, Furumido J, Iwahara N, et al. Objective Evaluation of Laparoscopic Surgical Skills in Wet-lab Training Based on Motion Analysis and Machine Learning. Langenbeck’s Arch. Surg. 2022; doi: 10.1007/s00423-022-02505-9 35394212 PMC9399206

[18] Vassiliou MC, Feldman LS, Andrew CG, Bergman S, Leffondré K, Stanbridge D, et al. A global assessment tool for evaluation of intraoperative laparoscopic skills. Am J Surg. 2005; 190(1):107–13. doi: 10.1016/j.amjsurg.2005.04.004 15972181

