# Supplementary material for: Automatic assessment of laparoscopic surgical skill competence based on motion metrics
Source: PLoS One. 2022 Nov 2;17(11):e0277105. doi: 10.1371/journal.pone.0277105 (PMC9629630; doi:10.1371/journal.pone.0277105)
Supplement: S1 Table — (DOCX) [file pone.0277105.s002.docx]

| Indices | | Unit | Definitions | Formulae |
| --- | --- | --- | --- | --- |
| General | |  |  |  |
| Operative time (Time) | | (s) | The time to complete a task. |  |
| Bimanual dexterity (BD) | |  | This index evaluates the coordinated movement between both hands, and is calculated from the relationship between the tip velocity of surgical instruments handled by both hands simultaneously [10]. | $\frac{\sum_{i=1}^{n} \left( v_{L}\left( i \right)- \bar{v_{L}} \right)\left( v_{R}\left( i \right)- \bar{v_{R}} \right)}{\sqrt{\sum_{i=1}^{n} \left( v_{L}\left( i \right)- \bar{v_{L}} \right)^{2}\sum_{i=1}^{n} \left( v_{R}\left( i \right)- \bar{v_{R}} \right)^{2}}}$ ,  Where $n$ is the total number of Mocap frames, and $v_{L}\left( i \right)$ and $v_{R}\left( i \right)$ are 3-dimensional tip velocities of instruments handled by left and right hands in frame $i$, respectively. $v_{L}\left( i \right)$ and $v_{R}\left( i \right)$ are calculated as follows: $v_{L}(i)=\left\Vert\boldsymbol{v}_{\mathbf{L}}\left( i \right) \right\Vert, v_{R}(i)=\left\Vert\boldsymbol{v}_{\mathbf{R}}\left( i \right) \right\Vert$. Here, $\bar{v_{L}}$ and $\bar{v_{R}}$ are averages of $v_{L}\left( i \right)$ and $v_{R}\left( i \right)$ in the measured task. |
| Ratio of frequency of opening/closing both forceps (ROB) | |  | The ratio of the opening/closing frequency of forceps handled by the left and right hands during a dissection task. (Left hand: grasping forceps, right hand: scissors forceps) | $\frac{N_{OC\_grasper}}{N_{OC\_scissor}}$, where $N_{OC\_grasper}$ and $N_{OC\_scissor}$ are the opening/closing frequencies of grasping and scissor forceps, respectively. |
| Ratio of path length for both hands (RPLB) | |  | The ratio of the path lengths of the forceps tip position of both hands in the dissection task. (Left hand: grasping forceps, right hand: scissors forceps) | $\frac{\mathrm{PL}_{\mathrm{grasper}}}{\mathrm{PL}_{\mathrm{scissor}}}$, where $\mathrm{PL}_{\mathrm{grasper}}$ and $\mathrm{PL}_{\mathrm{scissor}}$ are path lengths of grasping and scissor forceps, respectively. |
| Average distance between both forceps when opening/closing (ADBO) | | (m) | The average distance between tip positions of instruments handled by both hands when opening and closing the grasping forceps in the dissection task. | $\frac{\sum_{i=1}^{N_{OC\_grasper}} \left\Vert\boldsymbol{p}_{\mathbf{tip}\left( \boldsymbol{grasper\_oc} \right)}(i)-\boldsymbol{p}_{\mathbf{tip}\left( \boldsymbol{scissor\_oc} \right)}\mathbf{(}i\mathbf{)} \right\Vert}{\mathrm{NOC}_{\mathrm{grasper}}}$,  where $\boldsymbol{p}_{\mathbf{tip}\left( \boldsymbol{grasper\_OC} \right)}\left( i \right)$ and $\boldsymbol{p}_{\mathbf{tip}\left( \boldsymbol{scissor\_oc} \right)}\left( i \right)$ are the tip positions of grasping and scissors forceps in the $i$ th opening/closing operation, respectively. |
| Average distance between both forceps (ADB) | | (m) | Average distance between tip positions of both forceps in the suturing/knotting task. | $\frac{\sum_{i=1}^{n} \left\Vert\boldsymbol{p}_{\mathbf{tip}\left( \mathbf{right} \right)}(i)-\boldsymbol{p}_{\mathbf{tip}\left( \mathbf{left} \right)}\mathbf{(}i\mathbf{)} \right\Vert}{n}$,  where $\boldsymbol{p}_{\mathbf{tip}\left( \mathbf{left} \right)}\mathbf{(}i\mathbf{)}$ and $\boldsymbol{p}_{\mathbf{tip}\left( \mathbf{right} \right)}\mathbf{(}i\mathbf{)}$ are the tip position of the left and right needle holder in frame $i$, respectively. |
| Forceps motion | |  |  |  |
| Path length (PL) | | (m) | Total movement of the tip of an instrument during the measured task. The tip trajectory that moved outside the box trainer is excluded from calculation. | $\sum_{i=1}^{n-1} \left\Vert\boldsymbol{p}_{\mathbf{tip}}\left( i+1 \right)-\boldsymbol{p}_{\mathbf{tip}}\left( i \right) \right\Vert$,  where, $\boldsymbol{p}_{\mathbf{tip}}\left( i \right)$ is the tip position of an instrument in frame $i$. The positional vector $\boldsymbol{p}_{\mathbf{tip}}$ is smoothed by the Savitzky-Golay filter. |
| Average velocity ($\bar{v}$) | | $\left( \mathrm{cm}/s \right)$ | Average tip velocity of an instrument. | $\frac{1}{n}\sum_{i=1}^{n} \left\Vert\frac{d}{dt}\boldsymbol{p}_{\mathrm{tip}}(i) \right\Vert$, where $\frac{d}{dt}\boldsymbol{p}_{\mathrm{tip}}(i)$ is calculated by the Savitzky-Golay filter. |
| Average acceleration ($\bar{a}$) | | $\left( \mathrm{cm}/{s^{2}} \right)$ | Average tip acceleration of an instrument. Acceleration is defined as the changing rate of velocity. | $\frac{1}{n}\sum_{i=1}^{n} \left\Vert\frac{d^{2}}{dt^{2}}\boldsymbol{p}_{\mathrm{tip}}(i) \right\Vert$, where $\frac{d^{2}}{dt^{2}}\boldsymbol{p}_{\mathrm{tip}}(i)$ is calculated by the Savitzky-Golay filter. |
| Average jerk ($\bar{j}$) | | $\left( \mathrm{cm}/{s^{3}} \right)$ | Average tip jerk of an instrument. Jerk is defined as the changing rate of acceleration, and it represents motion smoothness. | $\frac{1}{n}\sum_{i=1}^{n} \left\Vert\frac{d^{3}}{dt^{3}}\boldsymbol{p}_{\mathrm{tip}}(i) \right\Vert$, where $\frac{d^{3}}{dt^{3}}\boldsymbol{p}_{\mathrm{tip}}(i)$ is calculated by the Savitzky-Golay filter. |
| Distribution of working area　(Close/Near/Far zone) | Close | (%) | This index is calculated as the ratio of the path length moving around the target objects in a certain area (Close, Near, and Far) to the total path length. In the dissection task, the dissection area of the aorta was set as the target, and in the suturing/knotting task, the incised line of the kidney parenchyma was set as the target. The target objects were designated by putting the tip of forceps of both hands on the start/end point of the target for 5 s before conducting the experiment. | $\frac{\sum_{i=1}^{n-1} \left\Vert\boldsymbol{p}_{\mathrm{tip}}\left( i+1 \right)-\boldsymbol{p}_{\mathrm{tip}}\left( i \right) \right\Vert}{\mathrm{PL}}$ $=\left\{ i\in\left( 0,\ldots n-1 \right)\vert\left\Vert\left( \boldsymbol{p}_{\mathbf{start}}+l\frac{\boldsymbol{p}_{\mathbf{end}}\boldsymbol{-}\boldsymbol{p}_{\mathbf{start}}}{\left\Vert\boldsymbol{p}_{\mathbf{end}}\boldsymbol{-}\boldsymbol{p}_{\mathbf{start}} \right\Vert} \right)-\boldsymbol{p}_{\mathbf{tip}}\left( i+1 \right) \right\Vert<0.02 \right\} \left( \mathrm{cm} \right)$, where $\boldsymbol{p}_{\mathbf{start}}$ and $\boldsymbol{p}_{\mathbf{end}}$ are positions of start and end points of the target object, and $l$ is defined as follows: $l=\left\{ \begin{aligned} 0 \\ d \\ \left\Vert\boldsymbol{p}_{\mathbf{end}}\boldsymbol{-}\boldsymbol{p}_{\mathbf{start}} \right\Vert\end{aligned} \right.\begin{matrix} \left( d\leq0 \right) \\ \left( 0<d<\left\Vert\boldsymbol{p}_{\mathbf{end}}\boldsymbol{-}\boldsymbol{p}_{\mathbf{start}} \right\Vert\right) \\ \left( \left\Vert\boldsymbol{p}_{\mathbf{end}}\boldsymbol{-}\boldsymbol{p}_{\mathbf{start}} \right\Vert\leq d \right) \end{matrix} .$  Here, $d$ is calculated as follows: $d=\frac{\frac{\boldsymbol{p}_{\mathbf{end}}\boldsymbol{-}\boldsymbol{p}_{\mathbf{start}}}{\left\Vert\boldsymbol{p}_{\mathbf{end}}\boldsymbol{-}\boldsymbol{p}_{\mathbf{start}} \right\Vert}\boldsymbol{\cdot}\left( \boldsymbol{p}_{\mathbf{tip}}\left( i+1 \right)-\boldsymbol{p}_{\mathbf{start}} \right)}{\left\Vert\boldsymbol{p}_{\mathbf{end}}\boldsymbol{-}\boldsymbol{p}_{\mathbf{start}} \right\Vert^{2}}$. |
|  | Near |  |  | $\frac{\sum_{i=1}^{n-1} \left\Vert\boldsymbol{p}_{\mathbf{tip}}\left( i+1 \right)-\boldsymbol{p}_{\mathbf{tip}}\left( i \right) \right\Vert}{PL}$ $=\left\{ i\in\left( 0,\ldots n-1 \right)\vert0.02\leq\left\Vert\left( \boldsymbol{p}_{\mathbf{start}}+l\frac{\boldsymbol{p}_{\mathbf{end}}\boldsymbol{-}\boldsymbol{p}_{\mathbf{start}}}{\left\Vert\boldsymbol{p}_{\mathbf{end}}\boldsymbol{-}\boldsymbol{p}_{\mathbf{start}} \right\Vert} \right)-\boldsymbol{p}_{\mathbf{tip}}\left( i+1 \right) \right\Vert<0.04 \right\} \left( \mathrm{cm} \right).$ |
|  | Far |  |  | $\frac{\sum_{i=1}^{n-1} \left\Vert\boldsymbol{p}_{\mathbf{tip}}\left( i+1 \right)-\boldsymbol{p}_{\mathbf{tip}}\left( i \right) \right\Vert}{PL}$ $=\left\{ i\in\left( 0,\ldots n-1 \right)\vert0.04\leq\left\Vert\left( \boldsymbol{p}_{\mathbf{start}}+l\frac{\boldsymbol{p}_{\mathbf{end}}\boldsymbol{-}\boldsymbol{p}_{\mathbf{start}}}{\left\Vert\boldsymbol{p}_{\mathbf{end}}\boldsymbol{-}\boldsymbol{p}_{\mathbf{start}} \right\Vert} \right)-\boldsymbol{p}_{\mathbf{tip}}\left( i+1 \right) \right\Vert\right\} (cm)$. |
| Distribution of velocity　(Idle/Low/Middle/High/Very high) | Idle | (%) | This index calculated as the ratio of the number of frames whose instrument moves in a certain velocity band (Idle, Low, Middle, High, and Very high) to the total number of frames of the measured task. | $\frac{\left\vert n_{\mathrm{Idle}} \right\vert}{n} : n_{\mathrm{Idle}}=\left\{ i\in\left( 0,\ldots n \right) \vert0\leq v\left( i \right)<0.5 \right\} \left( \mathrm{cm}/s \right)$, where $v\left( i \right)$ is the tip velocity of an instrument in frame $i$. |
|  | Low |  |  | $\frac{\left\vert n_{\mathrm{Low}} \right\vert}{n} : n_{\mathrm{Low}}=\left\{ i\in\left( 0,\ldots n \right) \vert0.5\leq v\left( i \right)<2.0 \right\} \left( \mathrm{cm}/s \right)$. |
|  | Middle |  |  | $\frac{\left\vert n_{\mathrm{Middle}} \right\vert}{n} : n_{\mathrm{Middle}}=\left\{ i\in\left( 0,\ldots n \right) \vert2.0\leq v\left( i \right)<5.0 \right\} \left( \mathrm{cm}/s \right)$. |
|  | High |  |  | $\frac{\left\vert n_{\mathrm{High}} \right\vert}{n} : n_{\mathrm{High}}=\left\{ i\in\left( 0,\ldots n \right) \vert5.0\leq v\left( i \right)<12.0 \right\} \left( \mathrm{cm}/s \right)$. |
|  | Very high |  |  | $\frac{\left\vert n_{\mathrm{Veryhigh}} \right\vert}{n} : n_{\mathrm{Veryhigh}}=\left\{ i\in\left( 0,\ldots n \right) \vert5.0\leq v\left( i \right)<12.0 \right\} \left( \mathrm{cm}/s \right)$. |
| Depth path length (DPL) | | (m) | Total movement of the tip of an instrument along its sheath axis in the measured task. | $\sum_{i=1}^{n} \left\vert x_{L\left( i-1 \right)\mathrm{tip}}\left( i \right)-x_{L\left( i-1 \right)\mathrm{tip}}\left( i-1 \right) \right\vert$, where $x_{L\left( i-1 \right)\mathrm{tip}}\left( i \right)$ is tip position of an instrument on the X-axis in frame $i$, which is represented in the local coordinate system of the instrument in frame $\left( i-1 \right)$. The tip position of an instrument in frame $i$ $\boldsymbol{v}_{\mathbf{L}\left( \boldsymbol{i-1} \right)\mathbf{tip}}\left( i \right)=\left[ \begin{matrix} x_{L\left( i-1 \right)\mathrm{tip}}\left( i \right) & y_{L\left( i-1 \right)\mathrm{tip}}\left( i \right) & z_{L\left( i-1 \right)\mathrm{tip}}\left( i \right) \end{matrix} \right]$ is represented as follows:  $\boldsymbol{v}_{\mathbf{L}\left( \boldsymbol{i-1} \right)\mathbf{tip}}\left( i \right)=\boldsymbol{M}\left\{ \boldsymbol{v}_{\mathbf{Gtip}}\left( i \right)-\boldsymbol{v}_{\mathbf{Go}}\left( i-1 \right) \right\}$,  where $\boldsymbol{M}$ is the rotation matrix, calculated as follows: $\boldsymbol{M=}R_{x}\left( -\alpha_{i-1} \right)R_{y}\left( -\beta_{i-1} \right)R_{z}\left( -\gamma_{i-1} \right)$. Here, $R_{x}, R_{y}, and R_{z}$ are rotation matrices around X, Y, and Z-axes respectively, and $\alpha_{i-1} , \beta_{i-1}, and \gamma_{i-1}$ are attitude angles around X, Y, and Z-axes (Roll, Pitch, and Yaw) in frame $\left( i-1 \right)$ respectively. These angles are represented as Z-Y-X euler angles. |
| Depth velocity (DV) | | $\left( \mathrm{cm}/s \right)$ | Average velocity of the tip of an instrument on its sheath axis. | $\frac{1}{n}\sum_{i=1}^{n} \frac{\left\vert x_{L\left( i-1 \right)\mathrm{tip}}\left( i \right)-x_{L\left( i-1 \right)\mathrm{tip}}\left( i-1 \right) \right\vert}{DT}$ , where $DT$ (s) is the sampling period of the Mocap system. In this study, the sampling frequency was set to 30 Hz, and $DT$ was calculated as $DT=\frac{1}{30}=0.0333\cdots$ (s). |
| Number of opening/closing operations ($N_{\mathrm{OC}}$) | | (times) | The sum of repetitions of opening and closing the forceps jaw. “One repetition” is defined as “a series of opening and closing performed once”. |  |
| Average gripper rotation angle (AGRA) | | ($^{\circ}$) | Average rotation angle of the gripper of grasping forceps during the measured task. | $\frac{1}{n}\sum_{i=1}^{n} \theta_{\mathrm{gripper}}\left( i \right)$, where $\theta_{\mathrm{gripper}}\left( i \right)$ is the gripper rotation angle of an instrument in frame $i$*.* |
| Average attitude angle　(Roll/Pitch/Yaw) | Roll | ($^{\circ}$) | Average attitude angle (Roll/Pitch/Yaw) of an instrument during the measured task. These angles are represented as Z-Y-X Euler angles. | $\frac{1}{n}\sum_{i=1}^{n} \alpha\left( i \right)$. |
|  | Pitch |  |  | $\frac{1}{n}\sum_{i=1}^{n} \beta\left( i \right).$ |
|  | Yaw |  |  | $\frac{1}{n}\sum_{i=1}^{n} \gamma\left( i \right).$ |
| Angular length (AL-Roll/AL-Pitch/ Yaw) | AL-Roll | ($^{\circ}$) | The sum of changes in the attitude angle of an instrument. This index was defined for two items: the attitude angle around its sheath axis (Roll), and around the point of instrument insertion (Pitch/ Yaw). | $\sum_{i=1}^{n} \sqrt{\left( \alpha_{i}-\alpha_{i-1} \right)^{2}}$. |
|  | AL-Pitch/ Yaw |  |  | $\sum_{i=1}^{n} \sqrt{\left( \beta_{i}-\beta_{i-1} \right)^{2}+\left( \gamma_{i}-\gamma_{i-1} \right)^{2}}$. |
| Working area (WA) | | ($\mathrm{cm}^{2}$) | The moving area of the tip of forceps in a plane orthogonal to the target object (Y-Z plane in Figure 1). | $\left( y_{\mathrm{tip}\left( q=0.975 \right)}-y_{\mathrm{tip}\left( q=0.275 \right)} \right)\left( z_{\mathrm{tip}\left( q=0.975 \right)}-z_{\mathrm{tip}\left( q=0.275 \right)} \right)$, where $y_{\mathrm{tip}\left( q=0.975 \right)}$ and $y_{\mathrm{tip}\left( q=0.275 \right)}$ are the 97.5-percentile and 2.5-percentile of the tip position of an instrument in the Y-axis, respectively. $z_{\mathrm{tip}\left( q=0.975 \right)}$ and $z_{\mathrm{tip}\left( q=0.275 \right)}$ are calculated in the same way in the Z-axis. |
| Average inserting time (AIT) | | (s) | Average operating time to apply the clip using Hem-o-lok. This index is calculated as the time between inserting Hem-o-lok into and removing it from the box trainer. | $\frac{1}{n_{\mathrm{inserting}}}\sum_{i=1}^{n_{\mathrm{inserting}}} T_{\mathrm{inserting}}\left( i \right)$,  where $n_{\mathrm{inseting}}$ is the total number of insertions of Hem-o-lok in the measured task, and $T_{\mathrm{inserting}}\left( i \right)$ is the operating time of the $i$th insertion. |
