# Supplementary material for: Automatic assessment of laparoscopic surgical skill competence based on motion metrics
Source: PLoS One. 2022 Nov 2;17(11):e0277105. doi: 10.1371/journal.pone.0277105 (PMC9629630; doi:10.1371/journal.pone.0277105)
Supplement: S2 Table — (DOCX) [file pone.0277105.s003.docx]

| Classification algorithms | Description | Hyperparameters and grid search range |
| --- | --- | --- |
| Support Vector Machine (SVM) | SVM is a supervised machine-learning algorithm used for classification or regression. In SVM, support vectors that consist of the closest points of the border of two classes are used to calculate the hyperplane, which is the decision boundary. Using the RBF (Radial basis function) kernel, SVM can build a non-linear model and be applied to more complex classification problems. | Cost parameter C: $C=2^{-5}, 2^{-4}, \ldots, 2^{10}$Coefficient of RBF $\gamma$: $\gamma=2^{-20}, 2^{-19}, \ldots, 2^{10}$ |
| Principal component analysis-SVM (PCA-SVM) | This is the combined method of PCA and SVM. The principal component scores of the input data are used for the input of SVM. In addition, in the PCA process, the number of the principal component vector is reduced according to its cumulative proportion $P_{\mathrm{th}}$. | Threshold of the cumulative proportion $P_{\mathrm{th}}$: $P_{\mathrm{th}}=0.7, 0.8, 0.9$Note that the hyper parameter of SVM ($C, \gamma$) are the same as described above. |
| Gradient Boosting Decision Tree (GBDT) | GBDT is a supervised machine-learning algorithm using the decision tree. This method uses boosting, which is one of the ensemble methods, and the output is determined by the majority vote of classifiers. In order to reduce the model error in the learning process, the gradient method is used. By repeating a series of processes such as build a decision tree, estimation, and calculate error, GBDT can minimize errors and estimate class with high-level accuracy. | the number of decision trees built in the learning process $N_{\mathrm{tree}}:$  $N_{\mathrm{tree}}=10, 100, 1000, 2000,$  The number of leaves of a tree $N_{\mathrm{leave}}:$  $N_{\mathrm{leave}}=31, 63, 127$  the learning rate $\nu:$  $\nu=0.0001, 0.001, 0.01, 0.05, 0.1$In order to prevent overfitting, the max depth of the tree is set to 4. According to the sample size of the input data, the minimum number of samples in a child node is set to 3. |
