# Supplementary material for: Automatic assessment of laparoscopic surgical skill competence based on motion metrics
Source: PLoS One. 2022 Nov 2;17(11):e0277105. doi: 10.1371/journal.pone.0277105 (PMC9629630; doi:10.1371/journal.pone.0277105)
Supplement: S3 Table — (DOCX) [file pone.0277105.s004.docx]

| Indices | Novices (experience: 0-9), n=24 (N) | Intermediates (experience: 10-49), n=24 (I) | Experts (experience: 50-), n=41 (E) | *p*-value | N-I | I-E | N-E |
| --- | --- | --- | --- | --- | --- | --- | --- |
| *General* |  |  |  |  |  |  |  |
| Operative time (s) | 2303.56 (2004.47 to 2719.92) | 1200.41 (963.45 to 1487.73) | 997.57 (803.14 to 1219.03) | <0.0001 | <0.0001 | 0.0255 | <0.0001 |
| BD | 0.01 (-0.01 to 0.02) | 0.01 (0.00 to 0.02) | 0.02 (0.01 to 0.04) | 0.0386 | 0.3956 | 0.1606 | 0.0114 |
| ROB | 0.28 (0.19 to 0.47) | 0.18 (0.14 to 0.28) | 0.20 (0.12 to 0.27) | 0.0324 | 0.0319 | 0.8029 | 0.0146 |
| RPLB | 0.64 (0.51 to 0.72) | 0.57 (0.50 to 0.66) | 0.56 (0.49 to 0.63) | 0.0623 |  |  |  |
| ADBO | 0.04 (0.03 to 0.05) | 0.04 (0.03 to 0.05) | 0.04 (0.03 to 0.06) | 0.0916 |  |  |  |
| *Grasping forceps* |  |  |  |  |  |  |  |
| PL (m) | 22.47 (17.90 to 24.94) | 11.46 (7.43 to 13.45) | 8.20 (6.73 to 10.94) | <0.0001 | <0.0001 | 0.0388 | <0.0001 |
| $\bar{v}$ (cm/s) | 0.94 (0.80 to 1.13) | 0.91 (0.70 to 1.00) | 0.85 (0.78 to 1.00) | 0.3194 |  |  |  |
| $\bar{a}$ ($cm/s^{2}$) | 2.48 (2.05 to 2.93) | 2.35 (1.81 to 2.69) | 2.22 (2.00 to 2.63) | 0.4065 |  |  |  |
| $\bar{j}$ ($cm/s^{3}$) | 17.86 (14.64 to 20.72) | 16.57 (12.33 to 18.73) | 15.99 (14.58 to 19.41) | 0.4580 |  |  |  |
| Close zone (%) (<2.0 cm) | 35.65 (22.98 to 40.14) | 36.38 (28.27 to 44.40) | 28.15 (22.80 to 38.87) | 0.2437 |  |  |  |
| Near zone (%) (2.0-4.0 cm) | 46.38 (39.01 to 51.63) | 43.81 (39.40 to 48.66) | 45.88 (35.91 to 51.78) | 0.6661 |  |  |  |
| Far zone (%) (>4.0 cm) | 16.75 (6.64 to 29.90) | 16.76 (5.84 to 25.16) | 17.21 (8.38 to 34.35) | 0.4938 |  |  |  |
| Idletime (%) (<0.5 cm/s) | 52.03 (48.25 to 57.17) | 57.60 (52.82 to 64.98) | 56.88 (50.68 to 61.84) | 0.0649 |  |  |  |
| Low (%) (0.5-2.0 cm/s) | 32.83 (30.16 to 37.17) | 29.96 (26.55 to 32.49) | 32.44 (28.70 to 38.25) | 0.0180 | 0.0063 | 0.0190 | 0.7512 |
| Middle (%) (2.0-5.0 cm/s) | 10.84 (8.61 to 13.94) | 9.00 (6.79 to 12.84) | 9.13 (7.71 to 11.24) | 0.1067 |  |  |  |
| High (%) (5.0-12.0 cm/s) | 2.09 (0.91 to 3.22) | 2.02 (1.12 to 2.67) | 1.84 (1.10 to 2.30) | 0.7496 |  |  |  |
| Very high (%) (>12.0 cm/s) | 0.06 (0.01 to 0.09) | 0.05 (0.00 to 0.11) | 0.03 (0.00 to 0.08) | 0.6636 |  |  |  |
| DPL (m) | 7.14 (5.84 to 9.80) | 3.34 (1.82 to 4.71) | 2.40 (1.99 to 3.25) | <0.0001 | <0.0001 | 0.1958 | <0.0001 |
| DV (cm/s) | 0.31 (0.25 to 0.45) | 0.24 (0.19 to 0.35) | 0.25 (0.19 to 0.36) | 0.0550 |  |  |  |
| $N_{\mathrm{oc}}$ | 97.00 (79.00 to 127.75) | 47.00 (28.75 to 58.50) | 38.00 (27.00 to 57.00) | <0.0001 | <0.0001 | 0.5683 | <0.0001 |
| ATA ( ˚) | 12.60 (-7.65 to 74.39) | 17.06 (-19.40 to 69.26) | 32.75 (-40.08 to 101.17) | 0.9644 |  |  |  |
| Roll ( ˚) | -29.56 (-47.27 to 0.78) | -32.98 (-47.37 to -15.16) | -43.86 (-57.36 to -36.25) | 0.0255 | 0.4932 | 0.0316 | 0.0228 |
| Pitch ( ˚) | -28.04 (-29.48 to -23.91) | -28.00 (-29.35 to -26.14) | -29.91 (-32.94 to -25.94) | 0.0847 |  |  |  |
| Yaw ( ˚) | -42.11 (-46.46 to -36.97) | -38.51 (-44.41 to -33.65) | -38.93 (-42.05 to -35.13) | 0.2175 |  |  |  |
| AL-Roll ( ˚) | 8580.93 (6479.92 to 11206.97) | 4270.21 (2846.00 to 5201.74) | 3420.77 (2706.51 to 4863.96) | <0.0001 | <0.0001 | 0.3760 | <0.0001 |
| AL-PitchYaw ( ˚) | 8334.87 (6844.56 to 9754.17) | 4444.37 (2797.98 to 5271.90) | 3212.22 (2372.91 to 4480.75) | <0.0001 | <0.0001 | 0.0523 | <0.0001 |
| Working area ($cm^{2}$) | 26.20 (18.43 to 40.32) | 23.05 (14.78 to 28.38) | 22.57 (15.94 to 28.71) | 0.1890 |  |  |  |
| *Scissors* |  |  |  |  |  |  |  |
| PL (m) | 32.82 (28.02 to 41.00) | 17.88 (14.26 to 21.39) | 15.06 (13.35 to 17.64) | <0.0001 | <0.0001 | 0.0415 | <0.0001 |
| $\bar{v}$ (cm/s) | 1.79 (1.55 to 2.11) | 1.94 (1.72 to 2.26) | 2.11 (1.90 to 2.44) | 0.0255 | 0.1923 | 0.1017 | 0.0129 |
| $\bar{a}$ ($cm/s^{2}$) | 4.88 (4.26 to 5.68) | 5.42 (4.73 to 6.36) | 6.06 (5.26 to 7.06) | 0.0072 | 0.1060 | 0.0961 | 0.0027 |
| $\bar{j}$ ($cm/s^{3}$) | 34.38 (30.17 to 42.35) | 40.71 (34.22 to 47.30) | 43.66 (39.42 to 53.53) | 0.0013 | 0.0646 | 0.0444 | 0.0005 |
| Close zone (%) (<2.0 cm) | 70.05 (57.75 to 77.50) | 79.72 (67.68 to 88.16) | 84.86 (79.17 to 90.57) | 0.0004 | 0.0374 | 0.0961 | <0.0001 |
| Near zone (%) (2.0-4.0 cm) | 28.84 (17.68 to 40.19) | 18.77 (10.44 to 27.81) | 13.45 (8.49 to 19.81) | 0.0016 | 0.0710 | 0.1235 | 0.0002 |
| Far zone (%) (>4.0 cm) | 2.92 (2.00 to 5.65) | 3.39 (2.57 to 4.27) | 3.49 (2.59 to 3.94) | 0.8942 |  |  |  |
| Idletime (%) (<0.5 cm/s) | 12.89 (9.84 to 15.55) | 11.60 (8.33 to 12.93) | 7.46 (6.22 to 12.06) | <0.0001 | 0.0972 | 0.0129 | <0.0001 |
| Low (%) (0.5-2.0 cm/s) | 51.96 (44.59 to 57.44) | 50.57 (45.81 to 57.14) | 49.17 (42.94 to 53.41) | 0.3994 |  |  |  |
| Middle (%) (2.0-5.0 cm/s) | 31.65 (24.12 to 36.67) | 32.12 (28.94 to 38.40) | 37.99 (31.77 to 42.12) | 0.0108 | 0.2465 | 0.0715 | 0.0041 |
| High (%) (5.0-12.0 cm/s) | 2.45 (1.27 to 5.61) | 4.26 (2.31 to 6.65) | 5.07 (2.96 to 7.75) | 0.0754 |  |  |  |
| Very high (%) (>12.0 cm/s) | 0.04 (0.02 to 0.13) | 0.10 (0.05 to 0.17) | 0.14 (0.05 to 0.22) | 0.0617 |  |  |  |
| DPL (m) | 19.27 (15.59 to 22.94) | 10.43 (9.55 to 13.07) | 9.18 (8.01 to 10.43) | <0.0001 | <0.0001 | 0.0176 | <0.0001 |
| DV (cm/s) | 0.99 (0.78 to 1.18) | 1.09 (1.02 to 1.29) | 1.20 (1.06 to 1.35) | 0.0210 | 0.0710 | 0.3137 | 0.0072 |
| $N_{\mathrm{oc}}$ | 357.00 (255.25 to 431.75) | 236.00 (170.75 to 278.00) | 216.00 (155.00 to 253.00) | 0.0002 | 0.0013 | 0.3632 | <0.0001 |
| ATA ( ˚) | -52.32 (-68.06 to -29.43) | -27.99 (-75.20 to 6.22) | -51.47 (-72.44 to -16.82) | 0.7812 |  |  |  |
| Roll ( ˚) | 5.34 (-10.32 to 20.86) | 13.13 (-2.87 to 21.28) | 5.92 (-9.57 to 16.80) | 0.6213 |  |  |  |
| Pitch ( ˚) | 29.66 (27.85 to 31.55) | 30.84 (28.84 to 32.55) | 29.45 (27.34 to 31.55) | 0.5177 |  |  |  |
| Yaw ( ˚) | -53.72 (-57.43 to -50.83) | -54.06 (-58.05 to -50.06) | -57.17 (-61.65 to -50.13) | 0.2442 |  |  |  |
| AL-Roll ( ˚) | 15222.05 (14426.55 to 18495.22) | 8986.20 (7311.79 to 11080.98) | 7474.53 (6387.55 to 10170.20) | <0.0001 | <0.0001 | 0.1958 | <0.0001 |
| AL-PitchYaw ( ˚) | 23084.70 (20297.30 to 25229.25) | 13156.80 (10541.73 to 15421.62) | 10708.80 (9538.29 to 13780.60) | <0.0001 | <0.0001 | 0.0459 | <0.0001 |
| Working area ($\mathrm{cm}^{2}$) | 12.93 (7.68 to 17.41) | 8.89 (7.35 to 10.72) | 8.08 (6.97 to 10.02) | 0.0014 | 0.0077 | 0.4373 | 0.0003 |
| *Hem-o-lok* |  |  |  |  |  |  |  |
| PL (m) | 5.96 (4.69 to 6.75) | 4.55 (4.08 to 6.11) | 4.54 (3.66 to 5.24) | 0.0045 | 0.0415 | 0.3072 | 0.0009 |
| $\bar{v}$ (cm/s) | 3.01 (2.60 to 3.28) | 4.02 (3.32 to 4.54) | 4.03 (3.33 to 4.74) | <0.0001 | <0.0001 | 0.6033 | <0.0001 |
| $\bar{a}$ ($cm/s^{2}$) | 6.03 (5.10 to 6.73) | 8.44 (7.02 to 9.65) | 8.53 (7.17 to 10.76) | <0.0001 | <0.0001 | 0.8134 | <0.0001 |
| $\bar{j}$ ($cm/s^{3}$) | 34.96 (29.87 to 42.52) | 48.12 (39.66 to 54.08) | 45.21 (38.85 to 55.25) | 0.0004 | 0.0002 | 0.9731 | 0.0005 |
| Close zone (%) (<2.0 cm) | 19.11 (12.87 to 27.08) | 19.57 (12.00 to 22.90) | 19.46 (15.93 to 26.39) | 0.4168 |  |  |  |
| Near zone (%) (2.0-4.0 cm) | 27.26 (25.79 to 30.48) | 28.09 (25.18 to 30.36) | 23.35 (18.61 to 25.37) | 0.0003 | 0.7207 | 0.0024 | 0.0002 |
| Far zone (%) (>4.0 cm) | 55.78 (48.54 to 59.46) | 58.69 (52.80 to 65.14) | 60.73 (54.21 to 66.61) | 0.0983 |  |  |  |
| Idletime (%) (<0.5 cm/s) | 18.68 (15.11 to 23.62) | 15.18 (10.25 to 18.32) | 11.19 (8.86 to 16.24) | <0.0001 | 0.0128 | 0.1046 | <0.0001 |
| Low (%) (0.5-2.0 cm/s) | 48.28 (44.87 to 52.15) | 45.32 (43.02 to 49.00) | 45.55 (42.27 to 49.28) | 0.1806 |  |  |  |
| Middle (%) (2.0-5.0 cm/s) | 16.34 (14.49 to 21.30) | 19.34 (17.60 to 23.88) | 22.12 (18.68 to 24.68) | 0.0066 | 0.0890 | 0.2005 | 0.0014 |
| High (%) (5.0-12.0 cm/s) | 8.65 (7.15 to 9.99) | 10.71 (8.46 to 13.98) | 12.02 (8.45 to 13.92) | 0.0044 | 0.0128 | 0.6318 | 0.0013 |
| Very high (%) (>12.0 cm/s) | 5.99 (4.29 to 7.29) | 8.74 (6.58 to 9.94) | 7.75 (5.86 to 10.93) | 0.0033 | 0.0007 | 0.8134 | 0.0053 |
| DPL (m) | 3.47 (3.11 to 4.15) | 3.29 (2.72 to 3.66) | 3.07 (2.39 to 3.58) | 0.0811 |  |  |  |
| DV (cm/s) | 1.68 (1.58 to 1.97) | 2.32 (2.10 to 2.75) | 2.55 (2.25 to 2.98) | <0.0001 | <0.0001 | 0.1958 | <0.0001 |
| Roll ( ˚) | 26.88 (0.95 to 44.61) | 10.53 (-5.82 to 37.82) | 16.64 (1.35 to 43.72) | 0.7116 |  |  |  |
| Pitch ( ˚) | 34.42 (31.28 to 35.58) | 32.65 (31.24 to 34.59) | 32.64 (30.39 to 35.45) | 0.2924 |  |  |  |
| Yaw ( ˚) | -49.58 (-52.27 to -47.24) | -47.19 (-51.04 to -45.32) | -47.17 (-52.82 to -45.55) | 0.5302 |  |  |  |
| AL-Roll ( ˚) | 2009.69 (1593.06 to 3012.70) | 1692.16 (1142.77 to 2586.97) | 1549.23 (1216.00 to 2153.65) | 0.1241 |  |  |  |
| AL-PitchYaw ( ˚) | 1286.88 (936.97 to 1522.45) | 1091.79 (898.57 to 1291.99) | 966.88 (743.81 to 1202.28) | 0.0478 | 0.1060 | 0.4137 | 0.0169 |
| Working area ($\mathrm{cm}^{2}$) | 77.74 (52.15 to 107.61) | 70.96 (39.63 to 92.72) | 49.80 (42.62 to 91.84) | 0.0861 |  |  |  |
| AIT (s) | 17.40 (14.23 to 19.18) | 11.33 (9.07 to 12.97) | 9.64 (8.70 to 11.32) | <0.0001 | <0.0001 | 0.0831 | <0.0001 |

| Indices | Novices (experience: 0-9), n=24 | Intermediates (experience: 10-49), n=24 | Experts (experience: 50-), n=41 | p-value | N-I | I-E | N-E |
| --- | --- | --- | --- | --- | --- | --- | --- |
| *General* |  |  |  |  |  |  |  |
| Operative time (s) | 650.08 (382.89 to 977.02) | 317.72 (252.37 to 412.55) | 263.29 (222.36 to 311.10) | <0.0001 | <0.0001 | 0.0204 | <0.0001 |
| BD | 0.07 (0.01 to 0.17) | 0.15 (0.08 to 0.22) | 0.21 (0.13 to 0.30) | 0.0056 | 0.0930 | 0.1411 | 0.0014 |
| ADB | 0.03 (0.03 to 0.03) | 0.03 (0.03 to 0.03) | 0.03 (0.03 to 0.03) | 0.5448 |  |  |  |
| *Right needle holder* |  |  |  |  |  |  |  |
| PL (m) | 22.47 (17.90 to 24.94) | 11.46 (7.43 to 13.45) | 8.20 (6.73 to 10.94) | <0.0001 | 0.0011 | 0.0237 | <0.0001 |
| $\bar{v}$ (cm/s) | 0.94 (0.80 to 1.13) | 0.91 (0.70 to 1.00) | 0.85 (0.78 to 1.00) | 0.0002 | 0.0059 | 0.1820 | <0.0001 |
| $\bar{a}$ ($cm/s^{2}$) | 2.48 (2.05 to 2.93) | 2.35 (1.81 to 2.69) | 2.22 (2.00 to 2.63) | 0.0018 | 0.0128 | 0.4699 | 0.0003 |
| $\bar{j}$ ($cm/s^{3}$) | 17.86 (14.64 to 20.72) | 16.57 (12.33 to 18.73) | 15.99 (14.58 to 19.41) | 0.0164 | 0.0243 | 0.9946 | 0.0058 |
| Close zone (%) (<2.0 cm) | 35.65 (22.98 to 40.14) | 36.38 (28.27 to 44.40) | 28.15 (22.80 to 38.87) | 0.4045 |  |  |  |
| Near zone (%) (2.0-4.0 cm) | 46.38 (39.01 to 51.63) | 43.81 (39.40 to 48.66) | 45.88 (35.91 to 51.78) | 0.3453 |  |  |  |
| Far zone (%) (>4.0 cm) | 16.75 (6.64 to 29.90) | 16.76 (5.84 to 25.16) | 17.21 (8.38 to 34.35) | 0.1424 |  |  |  |
| Idletime (%) (<0.5 cm/s) | 52.03 (48.25 to 57.17) | 57.60 (52.82 to 64.98) | 56.88 (50.68 to 61.84) | 0.0493 | 0.3007 | 0.2103 | 0.0156 |
| Low (%) (0.5-2.0 cm/s) | 32.83 (30.16 to 37.17) | 29.96 (26.55 to 32.49) | 32.44 (28.70 to 38.25) | 0.0001 | 0.0010 | 0.6512 | <0.0001 |
| Middle (%) (2.0-5.0 cm/s) | 10.84 (8.61 to 13.94) | 9.00 (6.79 to 12.84) | 9.13 (7.71 to 11.24) | 0.0028 | 0.0287 | 0.3008 | 0.0006 |
| High (%) (5.0-12.0 cm/s) | 2.09 (0.91 to 3.22) | 2.02 (1.12 to 2.67) | 1.84 (1.10 to 2.30) | <0.0001 | 0.0010 | 0.2585 | <0.0001 |
| Very high (%) (>12.0 cm/s) | 0.06 (0.01 to 0.09) | 0.05 (0.00 to 0.11) | 0.03 (0.00 to 0.08) | 0.1848 |  |  |  |
| DPL (m) | 7.14 (5.84 to 9.80) | 3.34 (1.82 to 4.71) | 2.40 (1.99 to 3.25) | <0.0001 | 0.0008 | 0.0694 | <0.0001 |
| DV (cm/s) | 0.31 (0.25 to 0.45) | 0.24 (0.19 to 0.35) | 0.25 (0.19 to 0.36) | 0.0011 | 0.0172 | 0.1202 | 0.0003 |
| Roll ( ˚) | -29.56 (-47.27 to 0.78) | -32.98 (-47.37 to -15.16) | -43.86 (-57.36 to -36.25) | 0.1785 |  |  |  |
| Pitch ( ˚) | -28.04 (-29.48 to -23.91) | -28.00 (-29.35 to -26.14) | -29.91 (-32.94 to -25.94) | 0.3296 |  |  |  |
| Yaw ( ˚) | -42.11 (-46.46 to -36.97) | -38.51 (-44.41 to -33.65) | -38.93 (-42.05 to -35.13) | 0.4433 |  |  |  |
| AL-Roll ( ˚) | 8580.93 (6479.92 to 11206.97) | 4270.21 (2846.00 to 5201.74) | 3420.77 (2706.51 to 4863.96) | 0.5116 |  |  |  |
| AL-PitchYaw ( ˚) | 15773.50 (3648.87 to 26120.62) | 5072.18 (1328.10 to 19827.10) | 1957.71 (1108.57 to 16599.90) | 0.0075 | 0.0533 | 0.2945 | 0.0018 |
| Working area ($cm^{2}$) | 38.52 (29.08 to 45.20) | 31.55 (28.18 to 38.44) | 35.12 (30.49 to 40.73) | 0.3126 |  |  |  |
| *Left needle holder* |  |  |  |  |  |  |  |
| PL (m) | 9.94 (6.11 to 17.40) | 5.92 (4.83 to 7.24) | 4.86 (4.12 to 5.64) | <0.0001 | 0.0008 | 0.0444 | <0.0001 |
| $\bar{v}$ (cm/s) | 1.65 (1.53 to 1.77) | 1.82 (1.63 to 2.01) | 1.91 (1.72 to 2.13) | 0.0012 | 0.0533 | 0.1202 | 0.0002 |
| $\bar{a}$ ($cm/s^{2}$) | 4.06 (3.75 to 4.55) | 4.59 (3.92 to 5.12) | 4.67 (4.22 to 5.25) | 0.0075 | 0.0778 | 0.2256 | 0.0017 |
| $\bar{j}$ ($cm/s^{3}$) | 28.59 (26.87 to 33.08) | 31.94 (26.74 to 36.56) | 32.20 (29.41 to 36.71) | 0.0461 | 0.2638 | 0.2945 | 0.0114 |
| Close zone (%) (<2.0 cm) | 32.50 (20.30 to 38.59) | 30.77 (25.94 to 38.67) | 27.25 (20.12 to 34.41) | 0.1711 |  |  |  |
| Near zone (%) (2.0-4.0 cm) | 48.54 (42.44 to 52.50) | 49.06 (46.33 to 53.58) | 53.28 (47.45 to 57.43) | 0.0333 | 0.5880 | 0.0540 | 0.0204 |
| Far zone (%) (>4.0 cm) | 20.34 (11.26 to 30.36) | 16.67 (11.36 to 23.68) | 19.73 (12.73 to 25.97) | 0.7963 |  |  |  |
| Idletime (%) (<0.5 cm/s) | 24.06 (20.31 to 26.51) | 23.37 (20.40 to 24.48) | 20.19 (15.57 to 23.65) | 0.0067 | 0.4308 | 0.0401 | 0.0024 |
| Low (%) (0.5-2.0 cm/s) | 45.45 (43.63 to 48.20) | 43.67 (40.28 to 47.54) | 45.09 (42.04 to 46.19) | 0.2639 |  |  |  |
| Middle (%) (2.0-5.0 cm/s) | 25.49 (22.12 to 27.43) | 27.47 (25.09 to 29.70) | 29.90 (26.54 to 32.16) | 0.0006 | 0.0890 | 0.0305 | 0.0002 |
| High (%) (5.0-12.0 cm/s) | 4.05 (2.85 to 5.48) | 5.44 (3.77 to 7.86) | 5.12 (4.12 to 7.72) | 0.0198 | 0.0507 | 0.6318 | 0.0053 |
| Very high (%) (>12.0 cm/s) | 0.04 (0.01 to 0.13) | 0.06 (0.00 to 0.10) | 0.01 (0.00 to 0.41) | 0.9333 |  |  |  |
| DPL (m) | 5.20 (3.04 to 8.58) | 3.00 (2.42 to 3.65) | 2.48 (2.15 to 2.87) | <0.0001 | 0.0005 | 0.0401 | <0.0001 |
| DV (cm/s) | 0.81 (0.73 to 0.89) | 0.89 (0.78 to 1.00) | 0.95 (0.88 to 1.04) | 0.0009 | 0.1060 | 0.0673 | 0.0001 |
| Roll ( ˚) | -29.30 (-33.41 to 27.98) | -28.25 (-33.04 to 99.34) | -31.13 (-35.31 to 87.56) | 0.6154 |  |  |  |
| Pitch ( ˚) | -24.42 (-26.34 to -21.96) | -25.08 (-26.13 to -22.36) | -25.95 (-27.04 to -24.02) | 0.0879 |  |  |  |
| Yaw ( ˚) | -33.62 (-42.71 to -17.96) | -30.45 (-36.78 to -0.24) | -34.31 (-37.22 to -16.68) | 0.4184 |  |  |  |
| AL-Roll ( ˚) | 2416.26 (1214.28 to 6659.58) | 1987.02 (969.68 to 3278.88) | 2035.30 (707.88 to 4460.43) | 0.3763 |  |  |  |
| AL-PitchYaw ( ˚) | 4374.54 (2861.28 to 10318.30) | 2204.48 (1171.85 to 4887.94) | 1888.29 (1008.17 to 3588.94) | 0.0042 | 0.0153 | 0.4699 | 0.0012 |
| Working area ($cm^{2}$) | 36.07 (29.95 to 41.34) | 36.43 (30.06 to 45.77) | 33.86 (26.47 to 42.86) | 0.5777 |  |  |  |

Median (Interquartile range)

N=Novices, I=Intermediates, E=Experts
